# Draft Genome Sequence of a Multidrug-Resistant *Pseudomonas aeruginosa* Strain Isolated from a Patient with a Urinary Tract Infection in Khartoum, Sudan

**DOI:** 10.1128/genomeA.00203-17

**Published:** 2017-04-20

**Authors:** Mohamed Hussain, Malik Suliman, Abdalla Ahmed, Hisham Altayb, Elamin Elneima

**Affiliations:** a Department of Pharmaceutical Microbiology, International University of Africa, Khartoum, Sudan; b Department of Pharmaceutics, Faculty of Pharmacy, University of Khartoum, Khartoum, Sudan; c Department of Microbiology, College of Medicine, Umm Al Qura University, Makkah, Saudi Arabia; d Department of Microbiology, Faculty of Medical Laboratory Sciences, Sudan University of Sciences and Technology, Khartoum, Sudan

## Abstract

*Pseudomonas aeruginosa* infection is difficult to treat due to the presence of antibiotic resistance determinants. Here, we report the genome sequence of a multidrug-resistant *P. aeruginosa* strain isolated from a patient with a urinary tract infection in 2015.

## GENOME ANNOUNCEMENT

*Pseudomonas aeruginosa* is an opportunistic Gram-negative, rod-shaped gammaproteobacterium that is frequently implicated in nosocomial infections and, to a lesser degree, in community-acquired infections (1). It has a natural resistance to different classes of antimicrobial agents, along with the ability to acquire resistance to all other treatment choices (2). The basic mechanisms of acquisition of drug resistance include the prevention of access to the drug binding site, presence of efflux pumps, modification in target enzymes, and inactivation of the antibiotics (3). The sizes of *P. aeruginosa* genomes range from 5.8 to 7.3 Mb, which comprise a core genome consisting of more than 4,000 genes plus a variable accessory gene pool (4).

Here, we announce the draft genome sequence of a strain of *P. aeruginosa*, isolated from the urine of a patient at the Military Hospital in Khartoum state, Sudan. Because it was resistant to a broad spectrum of antibiotics, like ciprofloxacin, gentamicin, ceftazidime, piperacillin, and meropenem, this strain was selected from among more than 200 isolates collected in 2015.

Genomic DNA from *P. aeruginosa* was isolated using a QIAamp DNA minikit from Qiagen (Hilden, Germany). The strain identity was then confirmed to the species level by sequencing the 16S rRNA region. Whole-genome sequencing was performed using Illumina HiSeq 2500 sequencing platform with 101-bp read length. The sequence data were filtered with a Phred score of >20 and *de novo* assembled using SeqMan NGen version 13.0.0 (https://www.dnastar.com/t-nextgen-seqman-ngen.aspx) resulting in 240 contigs with an *N*_50_ of 56 kb. The estimated genome size was 6 Mb with a G+C content of 64.6%. The largest contig size was 59,941 bp. Automated genome annotation was carried out by means of the NCBI Prokaryotic Genome Annotation Pipeline and predicted 6,373 protein-coding sequences, 64 RNA coding genes, 56 tRNAs, and four ncRNAs.

By using ResFinder (https://cge.cbs.dtu.dk/services/ResFinder), several antibiotic resistance genes were found in this genome, including the beta-lactamase resistance genes *bla*VEB-1, *bla*PAO, and *bla*OXA-50; the chloramphenicol resistance gene *cat*B7; the fosfomycin resistance gene *fos*A; the tetracycline resistance gene *tet*(G); and the aminoglycoside resistance genes *aph*_*(3′)*_*-*via, *aph*_*(3′)*_*-IIb*, and *aadA6*.

A detailed report of our isolate will be included in a future publication, along with a full comparative analysis involving multiple published *P. aeruginosa* genomes.

### Accession number(s).

This whole-genome shotgun project has been deposited at DDBJ/ENA/GenBank under the accession number MVDK00000000. The version described in this paper is the first version, MVDK01000000.
